# The Role of the NLRP3 Inflammasome in the Molecular and Biochemical Mechanisms of Cervical Ripening: A Comprehensive Review

**DOI:** 10.3390/cells13070600

**Published:** 2024-03-29

**Authors:** Wojciech Flis, Maciej W. Socha

**Affiliations:** 1 Department of Perinatology, Gynecology and Gynecologic Oncology, Faculty of Health Sciences, Collegium Medicum in Bydgoszcz, Nicolaus Copernicus University, Łukasiewicza 1, 85-821 Bydgoszcz, Poland; wflis@copernicus.gda.pl; 2 Department of Obstetrics and Gynecology, St. Adalbert’s Hospital in Gdańsk, Copernicus Healthcare Entity, Jana Pawła II 50, 80-462 Gdańsk, Poland

**Keywords:** parturition, cervical ripening, NLRP3 inflammasome, inflammation

## Abstract

The uterine cervix is one of the key factors involved in ensuring a proper track of gestation and labor. At the end of the gestational period, the cervix undergoes extensive changes, which can be summarized as a transformation from a non-favorable cervix to one that is soft and prone to dilation. During a process called cervical ripening, fundamental remodeling of the cervical extracellular matrix (ECM) occurs. The cervical ripening process is a derivative of many interlocking and mutually driving biochemical and molecular pathways under the strict control of mediators such as inflammatory cytokines, nitric oxide, prostaglandins, and reactive oxygen species. A thorough understanding of all these pathways and learning about possible triggering factors will allow us to develop new, better treatment algorithms and therapeutic goals that could protect women from both dysfunctional childbirth and premature birth. This review aims to present the possible role of the NLRP3 inflammasome in the cervical ripening process, emphasizing possible mechanisms of action and regulatory factors.

## 1. Introduction

Seemingly insignificant, the uterine cervix is the main factor ensuring the smooth course of both pregnancy and labor. During a normal pregnancy, the cervix is responsible for providing a physical and immunobiological barrier, ensuring unrestricted intrauterine fetal maturing. In the final stage of term gestation (or during premature delivery), profound changes occur in the cervical tissue, leading to extensive changes in cervical tissue composition. Macroscopically, these changes are manifested by transformation from rigid and tender to soft and prone to dilation. This change in the structure of the cervical tissue allows it to dilate properly during uterine contractions. Macroscopic changes in the cervix’s consistency result from several interrelated biochemical and molecular pathways leading to a total rearrangement of the extracellular matrix of the cervical tissue stroma. The set of these numerous intertwining metabolic pathways can be generally summarized as the cervical ripening process [1]. Cervical tissue remodeling is as prerequisite for proper parturition. As a result of the above processes, the cervix increases its softness and susceptibility, which allows labor to occur.

Despite the countless studies on cervical tissue metabolism, the whole process of cervical remodeling is poorly understood. The complex process of cervical ripening comprises a vast array of well-orchestrated biochemical pathways in which cooperation is required for the proper course of cervical remodeling. Numerous regulatory factors are known to have a significant impact on this process. These factors mainly include cytokines, metalloproteinases, prostaglandins, hormones, transcription factors, reactive oxygen species, etc. [2]. However, more and more attention has recently been assigned to the role of the local inflammatory response in cervical ripening. Inflammatory events occurring in cervical stroma include invasion of inflammatory cells (such as neutrophils and macrophages) and increased levels of inflammatory cytokines [2]. At the same time, these processes are strictly regulated both at the cellular and molecular levels [2,3].

Inflammasomes are a class of multimeric protein complexes that mediate the activation of potent inflammatory factors [4]. They are integral components of the innate immune response and actively participate in the development of the inflammatory cascade through their activation by inflammatory factors or stressors. Nucleotide-binding domain, leucine-rich-containing family, pyrin domain-containing-3 (NLRP3), which is one of the best-known and studied inflammasomes, greatly contributes to the inflammatory process by mediating the secretion of interleukin-1 (IL-1), which is a pivotal pro-inflammatory cytokine [5]. Numerous studies indicate the active involvement of the NLRP3 inflammasome in the development of the inflammatory response during pregnancy-associated complications [6]. However, whether the NLRP3 inflammasome is implicated in the cervical tissue remodeling process is largely unknown and poorly understood. Additionally, it is not fully understood what impact the NLRP3 inflammasome may have on cervical tissue biology.

In this review, we would like to discuss the role of the NLRP3 inflammasome in the process of cervical ripening, taking into account possible mechanisms of action and emphasizing its possible regulatory factors.

## 2. Uterine Cervix Biology

The uterine cervix can be divided into vaginal and supravaginal portions [7]. The cervical canal is a center structure of the cervix and is lined with a cylindrical epithelium with a single layer of cells producing significant amounts of mucus, containing ions, enzymes, mucin glycoproteins, and plasma proteins [7]. As gestation progresses, cervical epithelial cells proliferate significantly, leading to increased production of cervical mucus. Due to specific mucus formation, it is possible to provide an immunological barrier that ensures unimpeded fetal intrauterine development [7,8].

The uterine cervix is composed, in particular, of ground substance, connective tissue, and smooth muscle with the addition of cellular elements. The cervical structure is infiltrated by vast numbers of blood vessels, nerves, and lymphatic vessels. The cellular compartment of the cervix is composed mainly of fibroblasts, mast cells, and wandering cells [9].

The smooth muscle content of the cervical tissue accounts for approximately 15% of the dry mass of the cervix [9]. The distribution of muscle fibers along the cervix is extremely interesting. The area of the external cervical os is intertwined with muscle fibers, which constitute approximately 10% of the cervix, and these fibers are loosely dispersed in space. In turn, in the area of the internal os, muscle fibers are present in much larger numbers [9,10]. Additionally, fibers located around the internal cervical os undergo much more advanced spatial organization and form a characteristic sphincter-like pattern [10]. This specific spatial organization of the internal cervical os may suggest the inherence of the sphincter-like fiber system.

Ground substance, also known as the extracellular matrix (ECM), is the basic building block of the cervix [1]. The main components of the ECM are collagen types I and III, with a small percentage of collagen IV [1,11]. Collagen production is provided by fibroblasts, which actively participate in the proper metabolism of cervical tissue [11]. Interestingly, in the peripartum period, a significant decrease in the expression of type I collagen mRNA in cervical tissue can be observed [12]. The collagen fibers of the cervical stroma are connected to each other by covalent bonds [11]. The formation of covalent bonds enables the creation of a durable (degradation-resistant) three-dimensional structure, which ensures the stiffness of the cervix during pregnancy [11,13]. The cervical stroma is composed of glycosaminoglycans (GAGs), which contain vast amounts of sulfate groups [14]. The presence of a large amount of sulfate residues ensures high hydrophilic properties of the GAG molecule. The most important GAGs that build the cervical ECM include dermatan sulfate, chondroitin sulfate, heparan sulfate, and hyaluronan (HA) [14]. GAGs have the ability to covalently bond the protein core, leading to the formation of proteoglycans. Proteoglycans can subsequently bind free collagen fibers via their free anionic residue [13,14,15]. There are three main types of proteoglycans that make up the cervical ground substance: chondroitin (PG-L), decorin (PG-S2), and biglycan (PG-S1) [16]. The formation of such a three-dimensional structure composed of proteoglycans and collagen fibers enables the creation of a compact, stiff structure that is resistant to proteolytic degradation [11,12,13,14,15,16,17,18,19]. Taking the above into consideration, it seems that qualitative changes in proteoglycans or collagen arrangement may have profound effects on cervical tissue mechanics.

Elastin fibers also contribute to building the stroma of the cervix [9,20]. They consist of elastin polymers, which are cross-linked into more advanced spatial structures known as microfibrils [21]. In the non-pregnant stage, microfibrils show a long, organized, and well-organized structure. However, during gestation progression, elastic fibers’ concentration gradually decreases, and their structure becomes less organized and more randomly scattered in cervical tissue [21,22].

As mentioned earlier, during the perinatal period, the cervical tissue undergoes fundamental structural changes characterized as cervical ripening. These structural alterations can generally be described as a spatial remodeling of the cervical ECM. The descending effect of these changes is a significant loosening of the structure of the previously tightly packed cervix and an increase in the softness and compliance of the vaginal portion of the uterine cervix [23,24]. From a histological point of view, the main changes occurring in the cervix are the degradation of collagen and elastin fibers (accompanied by a decrease in total collagen concentration), increased water inflow (causing an increase in tissue hydration), increased HA synthesis, an increase in cervical cells’ apoptosis, and the occurrence of a local inflammatory response [9,11]. The whole process combines a vast range of interwoven (and interdependent) biochemical and molecular processes that are strictly controlled by regulatory factors.

During the gestational period, the synthesis of HA gradually increases, with the greatest increase in the perinatal period. Moreover, the transcription of genes encoding the hyaluronan synthase (HAS) enzyme increases throughout pregnancy—mainly HAS2 isoform [19,20]. In the perinatal period, HA remains the dominant GAG in cervical tissue [20]. An increased HA concentration in the cervical stroma, due to its hydrophilic properties, causes a significantly increased inflow of water into the ECM. As a result, an increased dispersion of collagen and elastin fibers in the cervix occurs during its remodeling [11,19,20,21,22,23].

The change that is most pronounced during cervical ripening is undoubtedly the decrease in total collagen concentration. This is achieved mostly by reducing the expression of collagen assembling genes and by increasing the enzymatic breakdown of collagen fibers [18,24,25,26,27,28].

**Figure 1:**
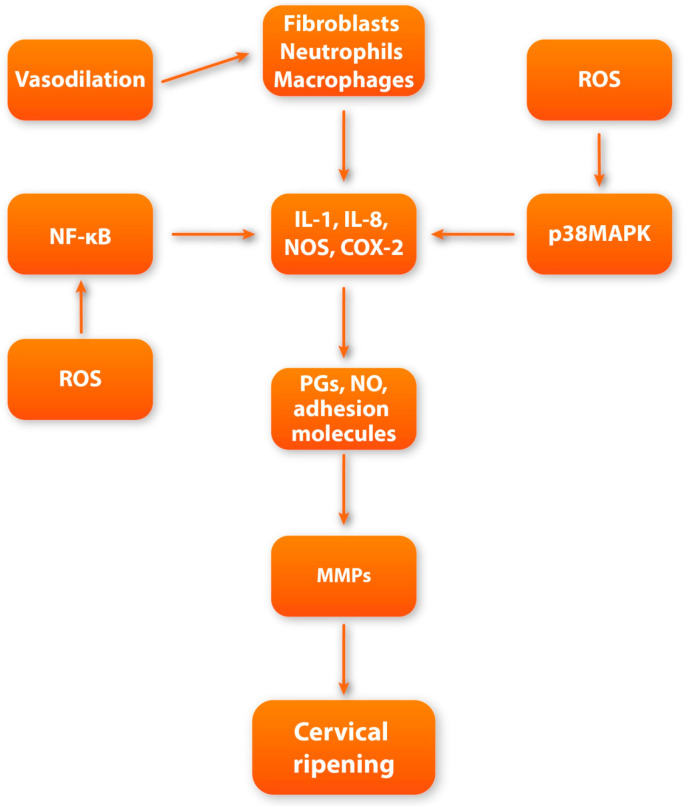
Scheme showing the overall course of biochemical and molecular pathways occurring during cervical ripening; ROS—reactive oxygen species; IL-1—interleukin-1; NF-kB—nuclear factor kappa-B; IL-18—interleukin-18; p38MAPK—p38 mitogen-activated protein kinase; PGs—prostaglandins; NO—nitric oxide; MMPs—metalloproteinases.

Enzymatic cleavage of collagen fibers is maintained by matrix metalloproteinases (MMPs) secreted mainly by fibroblasts, macrophages, neutrophils, and stromal cells [29]. MMPs are the main executors of the actual remodeling of the cervical ECM during its ripening [29,30]. In the perinatal period, there is a gradual increase in the activity and concentration of MMPs in the cervical tissue [31]. MMPs are synthesized and secreted in the form of prepro-enzymes (zymogens), which achieve their active form after enzymatic cleavage [32]. Activated MMPs can actively digest covalent bonds between collagen fibers, causing a change in ECM spatial organization—from tightly packed large multimers of collagen fibrils to smaller, loosely scattered bundles of fibers [33]. In turn, collagen fibers undergo spatial reorganization, leading to the significant softening of the cervix. Moreover, in addition to collagen fibers’ enzymatic breakdown, MMPs also have the ability to enzymatically cleave other ECM elements such as PG-S2, PG-L, and fibronectin [11]. The secretion and enzymatic activity of MMPs are closely regulated by other factors involved in pregnancy and parturition [34]. The expression of MMPs is enhanced by pro-inflammatory cytokines, nitric oxide (NO), prostaglandins (PGs), and growth factors [34,35]. In turn, their activity is suppressed by progesterone, retinoic acid, glucocorticoids, or specific tissue inhibitors of metalloproteinases (TIMPs) [36,37]. The cervical ripening process is under the strict influence of a variety of regulatory factors, which we will discuss in further sections of this review (Figure 1).

## 3. NLRP3 Inflammasome Biology

Inflammasomes are large cytoplasmic protein multimers that assemble in response to the detection of stress-associated or infectious stimuli [38]. They are pivotal for immune defenses against a variety of pathogens. Inflammasomes are involved in the immunological response against viral and bacterial infections [39,40]. Additionally, inflammasomes are crucial in maintaining cell homeostasis during stress-related events or environmental irritants [41]. Pattern-recognition receptors (PRRs) are a family of receptors that have the ability to be activated upon exposure to exogenous stimuli such as pathogens or a variety of endogenous factors [5]. PRRs recognize pathogen components known as pathogen-associated molecular patterns (PAMPs) or damage-associated molecular patterns (DAMPs), which are generated endogenously by cells subjected to a damaging factor [42]. Subsequently, those reactions trigger inflammatory response pathways in the cell environment. PRRs, apart from being present in human cells, can actively participate in inflammasome formation. Several PRRs have been shown to have the ability to form inflammasome multimers: the leucine-rich (LRR) repeat-containing protein family (NLRP1, NLRP3, etc.), pyrin, or absent-in-melanoma 2 protein (AIM 2) [43]. The names of the resulting inflammasomes arise from the names of the PRR sensor domains that constitute the inflammasome multimers during the inflammatory response, e.g., the NLRP3 inflammasome [5,42,43].

### 3.1. NLRP3 Inflammasome Structure

A typical inflammasome consists of a sensor protein (PRR), the adaptor apoptosis-associated speck-like protein (ASC) containing a caspase recruiting domain, and pro-inflammatory pro-caspase-1, which is responsible for further catalytic functions [44]. The formation of the inflammasome causes the activation of caspase-1, which subsequently cleaves the pro-inflammatory cytokines pro-interleukin-1β (pro-IL-1β) and pro-IL-18 into their mature, active forms—interleukin-1β (IL-1β) and interleukin-18 (IL-18) [45,46]. Subsequently, enzymatically cleaved interleukins participate in the inflammatory response. Apart from interleukins’ enzymatic activation, caspase-1 has the ability to trigger gasdermin D (GSDMD) activation, which participates in cell membrane pore formation, thereby activating the inflammatory form of cell death—pyroptosis [47]. The aim of pyroptosis is to remove potential pathogenic factors from the cell, allowing for their degradation by immunological factors. Additionally, as a result of pyroptosis, DAMPs and activated interleukins are removed from the cell, which may cause further activation of the inflammatory reaction in nearby cells [47,48,49,50].

The NLRP3 inflammasome is by far the most studied and well-known inflammasome, which is critical for proper host immune defense systems against pathogens such as bacterial, fungal, and viral infections. Additionally, NLRP3 has been linked with the pathogenesis of several diseases, such as Alzheimer’s disease or atherosclerosis [51]. NLRP3 is a tripartite molecule of the leucine-rich repeat (NLR) family, which consists of a central nucleotide-binding and oligomerization domain (NACHT or NOD), amino-terminal pyrin domain (PYD), and C-terminal leucine-rich repeat domain (LRR) [5,52]. The NLRP3 PYD domain is required for proper interaction with the PYD domain of the ASC protein. The NACHT domain has ATPase activity, which is responsible for the conformational change and oligomerization of the NLRP3 inflammasome [53]. The LRR domain has a receptor function and is responsible for external signal transduction [54]. The ASC protein of the forming inflammasome has a PYD domain (identical to the PYD domain of NLRP3) and a caspase activation and recruitment domain (CARD) that bonds to the corresponding CARD domain of caspase-1. Then, as a result of ATPase activation of the NACHT domain, oligomerization occurs, and a functional inflammasome is formed. Upon response to the stimuli, NLRP3 oligomerizes (leading to the spatial conformation of the multimer alteration) through NACHT domain interaction. Then, the ASC protein is recruited via PYD–PYD domain bonding. Assembled ASC complex recruits enable auto-cleavage and further activation of the caspase-1 enzyme [5,52,53,54].

### 3.2. NLRP3 Inflammasome Activation and Regulation

There are two possible routes to the formation and activation of the NLRP3 inflammasome—the canonical and non-canonical inflammasome assembly pathways [55]. The canonical pathway requires two signals for inflammasome activation—the priming and activation signals [56]. The priming signal is initiated by various PAMPs, IL-1β, Toll-like receptor (TLR) ligands, or tumor necrosis factor (TNF) [5]. The binding of specific PAMPs to their cell receptors induces the activation of nuclear factor-kappa-beta (NF-κB) with its subsequent translocation to the nucleus, where it enhances the transcription of genes encoding the NLRP3 protein (which belongs to the PRR group), pro- IL-1β, pro-IL-18, and gesdermin D [57,58,59]. Additional factors such as caspase-8 and FAS-associated death domain (FADD) are required for proper NF-κB nuclear translocation [60]. Apart from the correct priming signal, additional regulatory factors are required for proper NF-κB activation and translocation. Caspase-8 and FADD can interact with the IκB kinase (IKK) complex, which inhibits the transcriptional activity of NF-κB. Those regulating factors, by binding to IKK, cause the disinhibition of the NF-κB molecule, enabling its translocation to the nucleus, where it enhances NLRP3 and pro-IL-1 genes’ transcription [60]. The priming signal is necessary for NLRP3 to become more responsive to an activation signal. After the priming signal, the second stage occurs—the activation signal, which is triggered by a variety of PAMPs and DAMPs. The second signal step consists of NLRP3 oligomerization, ASC clustering, and caspase-1 bonding, leading to the full NLRP3 inflammasome assembly [61]. After the PYD domains of NLRP3 and ASC interact, the CARDS domain of ASC gains the ability to bond the CARD domain of pro-caspase-1. At the same time, the NACTH domain induces the oligomerization of the forming NLRP3 inflammasome. As a result, a functioning, mature NLRP3 inflammasome assembles [62]. Subsequently, after enzymatic self-cleavage, caspase-1 is released, leading to the activation and secretion of IL-18 and IL-1β [5,57,58,59,60]. Additionally, mature caspase-1 induces pyroptosis via gesdermin D activation. Caspase-1 cleaves GSDMD into N-term GDMD, which forms pores in the cell membrane, leading to the subsequent cytokines’ release from the cell [63,64,65]. Released cytokines can act as other DAMPs, leading to the activation and formation of new inflammasomes in neighboring cells. We believe that this two-phase pathway of inflammasome assembly and activation seems to be explained by macrophages’ biology, which are the main cells involved in the inflammatory reaction. The inheritance of NLPR3 activators is an insufficient signal for the overall assembly of the mature inflammasome [61]. Activated NF-κB enhances the NLRP3 expression, which is believed to be at levels inadequate to trigger inflammasome activation under physiological conditions [61]. However, while the primary signal has an effect on NLRP3 and IL-1β concentrations, it appears to have no effect on IL-18 and pro-caspase-1 concentrations [58]. This may suggest that the activation (second) signal is crucial for proper assembly and full activation of a functional NLRP3 inflammasome.

Various factors, mainly PAMPs and DAMPs, can induce an activation signal (second-stage signal) for NLRP3 inflammasome assembly. These factors do not appear to influence NLRP3 directly but through several common, overlapping biochemical pathways that lead to the activation of the NLRP3 inflammasome [5,11,66]. The activation of the NLRP3 inflammasome is stimulated by DAMPs, also known as alarmins, such as IL-1β, ATP, reactive oxygen species (ROS), high mobility group box-1 (HMGB1), and uric acid crystals [66]. PAMPs, meanwhile, refer to molecular fragments of pathogens such as bacterial RNA, bacterial DNA, fungi, and viral DNA [66,67,68,69].

Multiple molecular and biochemical cellular pathways that are activated by NLRP3 stimuli, including ionic flux, and ROS production have been shown to lead to NLRP3 inflammasome formation. Moreover, those substances can also act as alarmins and enhance the further NLRP3 priming signal, leading to the formation of inflammasomes in neighboring cells [5,62].

When a specific alarm signal reaches the cell, the primary signal is activated, and several biochemical and molecular pathways are triggered, inducing a second inflammasome activation signal. The NLRP3 stimuli can lead to an alteration in the ion potential of cells. These changes are mainly expressed by changes in the concentrations of calcium, potassium, chloride, and sodium ions [70]. When a damage signal occurs (e.g., PAMPs or DAMPs), the ATP-dependent ion channel P2RX7 is activated, which causes the efflux of K^+^ from the cell [70,71]. A decrease in the concentration of K^+^ in the cytoplasm causes the strengthening of the interaction between the never-in-mitosis gene A-related kinase 7 (NEK7) and NLRP3, leading to the activation of the NLRP3 inflammasome [62,72]. NEK7 belongs to the NIMA-related kinase (NEK proteins) serine/threonine kinase family and is indispensable for the NLRP3 inflammasome’s proper assembly [73]. The LRR domain of NLRP3 can bond with the catalytic domain of NEK7 in a kinase-dependent manner [74]. It seems that NEK7 is a crucial factor required for proper inflammasome activation. Recent studies report that caspase-1 and IL-1 secretion were suppressed in the absence of NEK7 [72]. Moreover, cells lacking NEK7 were unable to conduct proper ASC oligomerization [75]. This may suggest that despite the presence of a trigger signal, the lack of NEK7 activity results in the inability to properly assemble and activate the NLRP3 inflammasome. We believe that targeted anti-NEK7 treatment could prove effective in suppressing inflammasome assembly in a number of pregnancy-associated complications. However, this interesting topic requires further, in-depth research on the interrelationships between NEK7 and other factors regulating the inflammasome activation process.

At the same time, excess potassium ions cause damage to mitochondria, which increases ROS synthesis [76]. Additionally, the previously activated P2RX7 channel also enables the transport of calcium ions into the cell. The increase in Ca^2+^ in the cell also occurs through the activation of CASR receptors [77]. Ca^2+^ causes the activation of phospholipase C (PLC), which metabolizes phosphatidylinositol 4,5-bisphosphate (PIP2) to diacylglycerol (DAG) and 1,4,5-triphosphate (IP3). As a result, IP3, via binding to the endoplasmic reticulum (ER) membrane, causes the release of an additional portion of Ca^2+^ from the ER into the cytosol [78]. Excess calcium ions can cause direct formation of the NLPR3-ASC complex [78]. Additionally, overproduction of Ca^2+^ may lead to increased ROS production in the mitochondria [77]. Finally, a rapid change in the cell’s ionic potential may cause damage to the mitochondrial membrane, which will additionally translate into increased ROS production and the release of cardiolipin into the cytoplasm, which is an activator of apoptosis [61,69]. Additionally, the change in ionic potential also leads to damage to the lysosomal membrane, causing the release of cathepsin B into the cytoplasm, which has lysosomal protease activity [57]. Cathepsin B (as well as K^+^ and Ca^2+^) may directly activate specific kinases such as Janus kinases (JAKs) and Tat-associated kinases (TAKs), which can directly enhance the activation of the NLRP3 inflammasome [77,78,79,80,81,82].

Studies have revealed that knockdown of membrane chloride channels CLIC1 and CLIC4 impairs the transcription and secretion of pro-IL-1β, suggesting the active involvement of chlorine ions in the action of the inflammasome assembly [83,84]. CLICs are transmembrane channels responsible for Cl^−^ efflux [84]. Overproduction of ROS by mitochondria can induce the translocation of CLICs to cell membranes, which will lead to the removal of chloride ions from cells [85]. A decrease in the Cl^−^ concentration in cells leads to a direct increase in NEK7-NLRP3 interaction and subsequent assembly of the inflammasome [82,83]. Moreover, the decrease in Cl^−^ concentration induces oligomerization of ASC domains, which stimulates inflammasome assembly. However, this process does not occur in the absence of K^+^ efflux [82,83,84,85,86,87]. This may suggest that changes in ion concentration are interdependent and interact with each other in the process of inflammasome activation and assembly. However, this topic requires further research.

The non-canonical inflammasome activation pathway refers to the caspase-11-dependent inflammasome assembly [61]. This specific pathway occurs without priming and activating signals, as in the canonical pathway. This activation pathway is mainly associated with bacterial infections and contributes to the development of septic shock [88]. During the development of a bacterial infection, bacterial lipopolysaccharide (LPS) penetrates the cytoplasm, where it directly activates caspase-11 [89,90,91,92]. Caspase-11 then enzymatically cleaves GSDMD into N-GSDMD and C-GSDMD, which induces pyroptosis [93]. Additionally, active caspase-11 activates the release of potassium from the cell, which is a signal for the activation of the NLPR3 inflammasome, leading to the secretion of IL-1β [93,94,95]. Furthermore, cleaved N-GSDMD creates cell membrane pores during pyroptosis, facilitating IL-1 secretion [95,96,97].

In addition to the canonical and non-canonical activation pathways, there is also an alternative pathway for NLRP3 inflammasome activation [56]. This pathway requires only one activation signal (including LPS), which activates caspase-8. Active caspase-8 then directly activates the NLRP3 inflammasome [88,89,90,91,92,93].

## 4. Inflammatory Response and NLRP3 Inflammasome during Cervical Remodeling

As mentioned before, molecular and biochemical pathways occurring during cervical ripening greatly correlate with the local inflammatory reaction. The vast number of factors involved in triggering and maintaining the inflammatory response are also powerful and potent cervical ripening mediators [34].

### 4.1. Inflammation during Cervical Ripening and Its Regulatory Factors

Local vasodilatation and escalation of vascular permeability occur during cervical ripening [98]. The downstream effect of increased vascular permeability is the increased influx of leukocytes and water into the cervical ECM [98]. Subsequently, significant swelling of the cervical stroma can be observed. Referring to the research, the end of gestation is accompanied by a constant increase in the concentration of inflammatory cells both in the cervix and in other maternal–fetal compartments such as fetal membranes or the myometrium [99]. Among the inflammatory cells that infiltrate the cervical tissue at the end of pregnancy, the dominant cells are macrophages and neutrophils [100]. The rapid increase in granulocytes’ concentration is possibly due to the increased expression of chemokines and adhesion molecules. In the perinatal period, there is a significant increase in the expression of CXC chemokine ligand 8 (CXCL8) in cervical tissue, which acts as a strong neutrophilic chemoattractant [101]. Macrophages and neutrophils are among the most important factors involved in cervical ripening through their ability to secrete large amounts of MMPs, pro-inflammatory cytokines, prostaglandins, and ROS [11]. Due to their degranulation, the concentration of factors such as IL-1, IL-6, IL-8, nitric oxide (NO), prostaglandins (PGs), tumor necrosis factor (TNF), and adhesion molecules significantly increases [99]. All of the above molecules are strongly involved in triggering local inflammatory reactions and maintaining the proper cervical ripening process.

IL-1 is one of the key players and regulators of cervical ripening [102]. Studies suggest that intravaginal application of interleukin-1 leads to a great increase in cervical compliance, which is accompanied by an increased concentration of neutrophils and increased activity of neutrophilic proteases [103]. IL-1 appears to affect cervical ripening at multiple levels. First, IL-1 directly increases the expression of MMPs while inhibiting the activity of TIMPs, which leads to increased collagenolytic activity in cervical tissue [11,104,105]. Moreover, IL-1 can enhance the synthesis of other pro-inflammatory cytokines, such as TNF, IL-6, and IL-8 [106]. Finally, it has a high affinity for prostaglandin metabolism, causing inhibition of prostaglandin dehydrogenase (PGDH) and stimulating cyclooxygenase-2 (COX-2) expression. As a result, due to the action of IL-1, metabolism is inhibited, and the synthesis of prostaglandins in the cervical stroma is increased, resulting in a great increase in PG concentration [107,108].

Prostaglandins appear to influence the cervix through direct and indirect effects on the ECM. PGs increase the overall water content in the ECM and also stimulate fibroblasts to synthesize GAG. PGs also have the ability to regulate the activity of inflammatory cells [109,110]. By stimulating the secretion of endothelial adhesion molecule (ICAM-1), they enable adhesion with subsequent infiltration of leukocytes into the cervical tissue [109]. Additionally, prostaglandins inhibit the secretion of secretory leukocyte protease inhibitor (SLPI), which acts as a potent neutrophil inhibitor [111]. However, their most important impact in the context of cervical remodeling is the direct stimulation of the secretion and activity of MMPs, which leads to the enzymatic degradation of collagen fibers [112,113].

Another crucial regulator of the proper cervical ripening process, synthesized by macrophages and neutrophils, is IL-8. According to research, IL-8 synthesis increases significantly in cervical tissue at term [11,114]. IL-8 mostly influences cervical ripening by increasing the secretion of MMPs [115]. IL-8 not only enhances ECM remodeling but also greatly influences the development of a local inflammatory reaction. By directly stimulating vascular permeability and chemotactic activity, IL-8 leads to an increased influx and activation of neutrophils at the site of inflammation [116,117,118].

Another important factor that not only triggers but also facilitates the proper course of a vast array of biochemical pathways of cervical ripening is nitric oxide (NO) [119]. Nitric oxide is produced in a reaction mediated by nitric oxide synthase (NOS). The dominant form of NOS involved in cervical metabolic pathways is inducible nitric oxide synthase (iNOS). The main sources of NO in cervical tissue are macrophages and neutrophils that have the ability to express iNOS [11,120,121]. There is ample evidence regarding the direct involvement of NO in cervical ripening. In the research conducted, the local administration of NO donors led to the effective triggering of cervical ripening [122]. Meanwhile, the administration of NOS inhibitors led to the inhibition and delay of cervical ripening pathways in cervical tissue [123]. These data may suggest that nitric oxide is a potent inducer of cervical remodeling during the perinatal period. For instance, nitric oxide greatly affects the cervical stromal composition. Beyond this, the impact of NO on cervical tissue is mostly manifested by enhancing the activity of MMPs, which cleaves collagen cross-links [124]. The indirect effect is reflected by promoting the influx of leukocytes into the cervical ECM by inducing local vasodilation with additional stimulation of IL-8 secretion [125,126]. Moreover, NO can directly stimulate COX-2 expression in cervical cells, leading to an increase in the secretion of PGs [127,128,129].

Tumor necrosis factor alpha (TNF-α) belongs to the inflammatory cytokine group and resembles the pleiotropic effects of a variety of cells [130]. It is a homotrimeric molecule actively involved in the inflammatory response, which is generated mostly by activated macrophages [130]. Similarly to other pro-inflammatory factors, an increase in TNF secretion in the cervix is notable in the perinatal period [11]. The role of TNF-α in the local inflammatory reaction is mainly to stimulate the secretion of other pro-inflammatory cytokines such as IL-1, IL-6, and IL-8 [131]. Additionally, TNF-α is a known powerful stimulator of the activity of phospholipase A2, which is the main enzyme involved in the synthesis of PGs [130]. Moreover, TNF-α can induce the release of neutrophil granules, leading to an increase in the concentration of neutrophil proteases. Finally, TNF-α can also activate NF-κB, leading to increased expression of other pro-inflammatory factors involved in cervical remodeling [130,131,132]. Taking the above into account, it seems that TNF-α can actively participate in the alteration of the cervical ECM composition.

In addition to the above-mentioned molecules that directly participate in cervical ripening, it is also worth noting the factors that can regulate and connect numerous biochemical pathways that contribute to cervical ripening, such as nuclear factor kappa-B (NF-κB). NF-κB is a group of transcription factors that consist of molecules such as p50, cRel, RelA/p65, p52, and RelB [59]. NF-κB remains under the heavy influence of inflammatory factors, glucocorticoids, and progesterone, which are crucial factors involved in cervical ripening [133]. Activation of NF-κB results in its translocation to the nucleus, where it subsequently enhances specific inflammatory genes’ transcription. In turn, the expression of inflammatory reagents such as cytokines (IL-6, IL-8, and IL-1), iNOS, and PGs is enhanced. [133,134,135]. Subsequently, increased transcription of genes encoding the mentioned pro-inflammatory factors increases their expression in the cervical tissue, which leads to enhancement in remodeling during cervical maturation. Moreover, NF-κB activity is enhanced by ROS (formed by NADPH neutrophilic oxidase), PGs, and nitric oxide [133,134,135,136,137,138,139]. It seems that in addition to stimulating the expression of pro-inflammatory factors by NF-κB, these factors have the ability to additionally stimulate the activity of NF-κB. These interrelationships may suggest the presence of a regulatory circuit in which NF-κB is the key player. We believe that the increase in the concentration of pro-inflammatory mediators during the local inflammatory response in cervical tissue, by positively influencing NF-κB activity, leads to the maintenance of permanent NF-κB nuclear translocation, leading to the unimpeded transcription of NF-κB-dependent inflammatory genes [140].

Mitogen-activated protein kinases (MAPKs) are proline-directed threonine and serine protein kinases, which are pivotal players in the macrophage-mediated inflammatory response [141]. The p38 proteins are a class of MAPKs that are greatly expressed in macrophages. p38MAPKs can be classified into four subtypes: α (MAPK14), β (MAPK11), γ (MAPK12/ERK6), and δ (MAPK13/SAPK4), with the dominance of the α subtype [141,142]. They are activated by a vast array of factors, including ROS, cytokines, pathogens, growth factors, and estrogen [141].

The most well-known MAPK cascade consists of extracellular-signal-regulated (ERK) kinases MEK1/2 and ERK 1/2 (p42/p44) [141]. Phosphorylation of MAPKs leads to their activation via ERK kinases’ phosphorylation. Once activated, MAPKs predominantly regulate gene expression via phosphorylation of downstream transcription factors, which include the pro-inflammatory transcription factors activator protein (AP)-1 and NF-κB, leading to an increase in the expression of inflammation-related factors [141,142]. The evidence strongly suggests that apart from participating in the systemic macrophage-mediated inflammatory response, p38 is also involved in cervical ripening inflammatory pathways [143,144]. The involvement of p38MAPK in these pathways may be manifested through the increased expression of COX-2 and pro-inflammatory cytokines (such as IL-1, IL-8, and IL-6) [145,146,147,148,149]. Moreover, p38 can stimulate the secretion of endothelial vascular cell adhesion molecule-1 (VCAM-1), which is responsible for leukocyte inflow during the inflammatory response [149]. In addition, active p38MAPK may directly enhance the synthesis of MMPs (especially MMP-9), which are key players involved in the enzymatic cleavage of collagen fibers in the stroma of the cervix [149,150,151,152,153]. Taking the above into account, it seems that p38MAPK is an important regulatory factor that integrates several metabolic pathways and actively participates in triggering the inflammatory reaction during cervical ripening.

### 4.2. NLRP3 Inflammasome in the Cervical Inflammatory Response

The presented data clearly indicate the active involvement of the inflammatory process in the ripening of the cervix. It appears that the assembly and activation of the NLRP3 inflammasome may also contribute to the inflammatory response during cervical remodeling. According to the research, in a term pregnancy, there is an increase in the concentration of inflammasome-dependent caspase-1 in maternal–fetal compartments such as fetal membranes, uterine muscle, and cervical tissue [154,155]. These data are consistent with reports showing that IL-1β (which is the main product of the inflammasome) is elevated in cervical tissue in women during the physiological process of cervical ripening and preterm labor [11,156]. Interestingly, in addition to the increased concentration of caspase-1, a significant increase in the expression of the adapter protein ASC and GSDMD was also noted in the cervical tissue [157,158,159,160,161]. The presence of the ASC adapter protein may indicate the occurrence of inflammasome assembly and activation processes in the cervical ECM during its ripening. Moreover, the expression of GSDMD may suggest that inflammatory changes in the cervical stroma may also be accompanied by the process of pyroptosis.

Recent studies shed additional light on the active involvement of the inflammasome in the molecular pathways of cervical ripening. To investigate the contribution of the NLRP3 inflammasome to the cervical ripening and onset of preterm labor, Nlrp3-sufficient and Nlrp3-deficient mice were used, which were administered LPS during late gestation. Strikingly, Nlrp3-sufficient mice displayed increased cervical distension as compared with Nlrp3-deficient mice. LPS induced cervical dilation in Nlrp3-deficient mice but to a much lesser extent, indicating impaired cervical distension [162]. Moreover, Nlrp3-sufficient mice showed increased expression patterns of genes associated with cervical structural remodeling, such as proteoglycans and HAS genes, suggesting that NLRP3 may directly participate in cervical stromal spatial reorganization [162,163]. Finally, the expression of key inflammatory cytokines such as IL-1β, IL-6, IL-8, and TNF was greatly up-regulated in Nlrp3-sufficient mice [162,163,164]. These data clearly seem to indicate the active and direct involvement of the active NLRP3 inflammasome in the metabolic pathways of cervical ripening.

**Figure 2:**
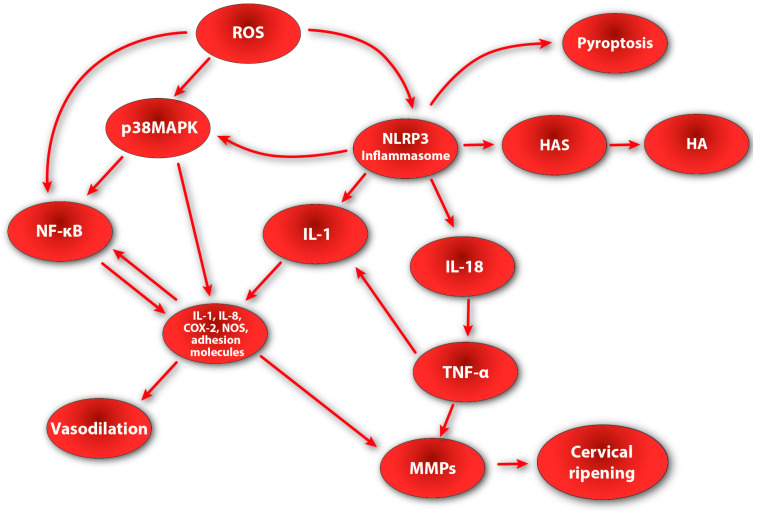
Diagram showing possible role of NLPR3 inflammasome in cervical ripening; NF-kB—nuclear factor kappa-B; ROS—reactive oxygen species; IL-1—interleukin-1; IL-18—interleukin-18; p38MAPK—p38 mitogen-activated protein kinase; PGs—prostaglandins; NO—nitric oxide; MMPs—metalloproteinases; COX-2—cyclooxygenase-2; TNF-*α*—tumor necrosis factor-*α*; HAS—hyaluronan synthase; HA—hyaluronan.

We believe that the influence of the active NLRP3 inflammasome on cervical ripening is multilevel. Firstly, due to its influence on the expression of genes encoding enzymes involved in cervical remodeling (such as hyaluronidase synthase), it directly participates in structural changes in the cervical ECM, which translates into an increased dispersion of collagen and elastin fibers in the cervical stroma. Moreover, the active NLRP3 inflammasome has the ability to synthesize and secrete IL-1β and IL-18. By increasing the concentration of IL-1β, the secretion of other pro-inflammatory cytokines and MMPs increases, as well as the synthesis of PGs in the cervical stroma. In turn, IL-18 directly stimulates the activity of TNF—a cytokine that regulates other biochemical pathways of cervical ripening [165]. As a result of the action of TNF, the synthesis of other pro-inflammatory cytokines is additionally enhanced, and the nuclear translocation of NF-κB is additionally enhanced, which translates into an increase in the secretion and synthesis of NO, PGs, and cytokines. It is also worth mentioning the possible relationship between the NLRP3 inflammasome and p38MAPK. In the research, increased NLRP3 inflammasome activity (due to LPS activation) with subsequent elevation of IL-1β and caspase-1 was accompanied by greater activation of p38MAPK [166,167]. These data may suggest that NLRP3 and p38MAPK activation pathways may be interdependent and intertwined. Taking into account the fact that p38MAPK can be up-regulated by a variety of mediators (e.g., cytokines or ROS), we believe that due to the activity of NLRP3 and the subsequent increase in the secretion of cytokines (mainly IL-1β), p38MAPK is activated, which additionally translates into an increase in the local inflammatory reaction (Figure 2).

Taking all of the above into consideration, we believe that the NLRP3 inflammasome is actively involved both directly and indirectly in the molecular and biochemical pathways during cervical ripening. It is certain that its components and products are present in the stroma of the cervix during its ripening and are involved in both the inflammatory reaction and structural remodeling of the cervix. However, it does not appear that NLRP3 plays a primary role in initiating and conducting cervical ripening. The conducted research shows that the lack of NLRP3 does not inhibit cervical ripening, but only significantly impairs it [162]. On the other hand, the local inflammatory response appears to be one of the most important biochemical processes occurring both in preterm birth as well as in cervical ripening. Therefore, the presence of an active NLRP3 inflammasome seems to be crucial for the proper and uninterrupted course of these reactions. Summing up, we believe that the role of the NLRP3 inflammasome in the biochemical pathways of cervical ripening is to regulate, connect, and mutually drive several pathways that result in the development of a normal local inflammatory response leading to cervical architectural alteration. However, this interesting topic requires further, thorough research.

## 5. NLRP3 Inflammasome and Reactive Oxygen and Nitrogen Species in Cervical Tissue

Reactive oxygen species are free radicals that can be classified as reactive molecules that contain an unpaired, additional electron. The dominant forms are reactive nitrogen species (RNS) and reactive oxygen species (ROS) [168]. ROS are produced in a variety of biochemical pathways throughout the cell and organelles, such as the mitochondrial respiratory chain, peroxisomes, and endoplasmic reticulum (ER) [169]. Under physiological conditions, ROS are continuously secreted in small amounts in cellular compartments and are actively involved in maintaining homeostasis of the internal environment. ROS and RNS actively participate in intracellular signaling, redox regulations, activation of protein kinases, ion channel opening, and protein modifications [170,171,172]. Moreover, ROS are also antimicrobial factors that can directly destroy microbial pathogens [172]. The variance in the concentration of antioxidant and pro-oxidant molecules can promote the occurrence of oxidative stress [173]. The ratio of the reactive molecules can be altered by an increase in the concentration of pro-oxidants or by a decrease in antioxidant mechanisms. Regardless of the mechanism, in both mentioned situations, an accumulation of highly reactive molecules can be observed [174]. During excessive ROS synthesis, the antioxidant systems become insufficient, which impairs proper cell functioning. Redundancy of ROS (under pathological conditions) leads to catastrophic events such as DNA damage, lipid peroxidation, and cellular membrane disruption [173,174].

The dominant free radicals involved in cell homeostasis are the superoxide anion, hydroxyl ion, and peroxynitrite (ONOO^−^), with a dominance of the superoxide anion. Physiological sources of the superoxide anion include the mitochondrial respiratory chain, nicotinamide adenine dinucleotide phosphate (NADPH) oxidase, and cytochrome P450 [175,176,177]. Reactive nitrogen species (RNS) include nitric oxide (NO), nitrogen dioxide (NO_2_), and ONOO^−^ [178]. NO is synthesized in the chemical reaction maintained by nitric oxide synthase (NOS), which requires NADPH as a cofactor and donor of additional electrons. NOS is an enzyme superfamily that consists of three isoforms: neuronal NOS (nNOS), inducible NOS (iNOS), and endothelial NOS (eNOS) [179]. Each of the isoforms is expressed in the cervical ECM, with a dominance of the inducible form [11,180,181]. Despite the fact that NO is a crucial molecule for proper cell homeostasis and cervical ripening, excessive secretion of NO can impair cell functionality [182].

The research clearly indicates the active involvement of reactive oxygen and nitrogen species in triggering and mediating the inflammatory response [183,184,185]. Moreover, it appears that ROS and RNS may also be actively involved in the biochemical and molecular pathways of cervical ripening. Referring to the research, ROS can directly stimulate the activity of p38MAPK in the cervical tissue, which is a strong mediator of the inflammatory reaction and also enhances the synthesis of other important factors involved in cervical ripening [145,186,187]. As mentioned earlier, stimulation of p38MAPK leads to increased expression of COX-2, pro-inflammatory cytokines, adhesion molecules, and MMPs [149]. Moreover, active p38MAPK has the ability to activate NF-κB, which leads to additional enhancement of the transcription of genes encoding factors such as NO, PGs, and pro-inflammatory cytokines [141]. In addition to activating NF-κB via p38MAPK (due to activation by ROS), reactive oxygen species can also directly stimulate the activation of NF-κB, which will result in intensified synthesis of the mentioned inflammatory reaction mediators. In its inactive form, NF-κB bonds with a specific NF-κB inhibitor, which can be expressed in three isoforms: IκBα, IκBβ, and IκBε [188]. NF-κB inhibitors are under strict regulation by IκB kinase (IKK). IKK can phosphorylate IκBα, subsequently leading to its degradation and allowing NF-κB to activate and translocate to the nucleus, where it becomes activated [188,189]. The excess ROS generated during the local inflammatory reaction can lead to the direct activation of protein kinase B (PKB) and NIK (NF-κB inducing kinase), whose role is to phosphorylate IKK. Once phosphorylated, IKK gains the ability to stimulate NF-κB. This leads to NF-κB activation, followed by an increase in the expression of factors regulating cervical ripening [190,191]. Moreover, pro-inflammatory cytokines involved in cervical remodeling can also stimulate ROS production. IL-1β and TNF (which are strong mediators of cervical ripening) can directly enhance the production of NADPH-oxidase-generated reactive oxygen species [192,193,194]. Subsequently, excess ROS may recruit other inflammatory mediators such as p38MAPK and NF-κB, which will lead to the boosting of inflammatory response in the cervical ECM. It appears that ROS and the NLRP3 inflammasome may also be associated with each other and act synergistically in the context of the development of a local inflammatory response. As mentioned earlier, both ROS and components of the NLRP3 inflammasome are present in cervical tissue during its remodeling period. Apart from the influence on NF-κB and p38MAPK, reactive oxygen species are potential inducers of NLRP3 inflammasome activation. Thioredoxin is believed to be involved in the activation of NLRP3 by ROS. Under resting conditions, thioredoxin-interacting protein (TXNIP) forms a complex with thioredoxin (TX) [195]. During excessive ROS production, an increased ROS concentration is sensed by the TXNIP-TX complex, leading to the dissociation of the complex. Then, liberated TXNIP can bind to the leucine-rich repeat of NLRP3, triggering the activation signal for NLPR3 inflammasome assembly [196,197].

Another possible mechanism for NLRP3 inflammasome activation by ROS is mitochondrial DNA (mtDNA) binding. Excessive production of ROS in mitochondria leads to the ROS binding to DNA strands, which leads to DNA damage and fragmentation. Then, free mtDNA fragments enter the cytoplasm, where they can directly initiate the assembly and activation of the NLRP3 inflammasome [196,198,199,200]. Activation of the NLRP3 inflammasome by ROS will result in increased secretion of caspase-1, IL-1β, and IL-18, which will translate into an increase in the inflammatory reaction and stimulation of further elements of its cascade, such as cytokines, PGs, MMPs, and NF-κB. Moreover, an active NLRP3 inflammasome can additionally enhance p38MAPK activity, which will further enhance the inflammatory response in cervical tissue. Furthermore, NLRP3-inflammasome-driven inflammation (induced by ROS) recruits inflammatory cells, including macrophages and neutrophils (whose concentration gradually increases in cervical tissue), which, in turn, leads to ROS production amplification, suggesting a feedback loop between ROS and the NLRP3 inflammasome. Taking the above into account, we believe that ROS not only directly participate in cervical ripening but are also responsible for the recruitment of additional, extremely important factors that regulate and stimulate the proper course of the local inflammatory reaction occurring in the cervical stroma. The presence of ROS seems to ensure the creation of a number of regulatory loops in which the components of the inflammatory process stimulate each other, leading to the intensification of changes occurring in the cervix.

## 6. Discussion

The main aim of this review was to provide the current state of knowledge of the possible involvement of the NLRP3 inflammasome in cervical ripening.

Cervical ripening is a derivative of the enzymatic breakdown of the extracellular matrix, mediated by a wide range of substances, which are strictly regulated by inflammatory and endocrine factors. There are numerous studies that describe the influence of specific factors on the cervical tissue in great detail. However, it has not yet been clearly postulated whether there is a single dominant factor or pathway that is responsible for triggering the complex biochemical pathways during cervical ripening. The data we described clearly indicate the active involvement of the NLRP3 inflammasome in the processes of cervical ripening. The NLRP3 inflammasome is intimately linked to almost every biochemical and molecular pathway involved in this complex process. After the activation and assembly of the NLRP3 inflammasome, IL-18 and IL-1β are secreted, which are potent mediators of cervical ripening. The secreted cytokines then activate a wide range of other mediators, such as MMP, NO, and PG. These substances, apart from directly affecting the remodeling of the cervix, may additionally intensify the inflammatory response. Starting from increasing vascular permeability and increasing the influx of inflammatory cells, through additional activation of NF-κB and p38MAPK, and ending with the activation of MMPs (decomposing collagen and elastin fibers), these substances lead to fundamental architectural changes in the cervix. Finally, substances whose synthesis is indirectly stimulated by the active NLRP3 inflammasome have the ability to additionally activate the NLRP3 inflammasome by acting as alarmins. Moreover, cytokines can enhance the production of ROS, which further amplify inflammasome activity. Taking the above into account, we believe that the NLRP3 inflammasome occupies a very important regulatory place in the processes of cervical ripening. Thanks to its activity, it is possible to connect and mutually stimulate molecular pathways dependent on cytokines, p38MAPK, NF-κB, and ROS.

In addition to its effects on the cervix, it appears that inflammasome activation may also influence uterine muscle contractility. As mentioned earlier, inflammasome components are present not only in the cervix but also in the myometrium during parturition. In previous work, the uterine tissue of Nlrp3-sufficient mice exhibited significantly increased expression of oxytocin receptors upon LPS injection as compared to the Nlrp3-deficient mice [163]. This may suggest that the assembly and activation of the NLRP3 inflammasome contributes to the increase in the expression of oxytocin receptors, which translates into increased contractility of the uterine myometrium during labor. When considering the function of the inflammasome, we should not forget the role of calcium ions in its activation. As mentioned earlier, increasing the concentration of calcium ions in the cell is one of the signals for the activation of the NLRP3 inflammasome. During labor, the contractility of the uterine myometrium depends largely on calcium ions. Both oxytocin and prostaglandins, by acting on their receptors, cause a significant increase in the concentration of calcium ions in the cytosol, which leads to the generation of muscle contraction [201,202]. We believe that an increase in the concentration of calcium ions in the cell (due to the interaction of PGs and oxytocin) additionally causes the activation of the NLRP3 inflammasome, which leads to an increase in the expression of oxytocin receptors and also increases the concentration of cytokines and caspase-1, which drives the development of a local inflammatory reaction. Moreover, in the research, the administration of MCC950, which is a specific inhibitor of the NLRP3 inflammasome, inhibited the occurrence of preterm birth and significantly reduced neonatal mortality [203,204]. Additionally, other studies conducted show that MCC950 could prove to be clinically safe and efficient for use in humans [205]. These data provide insights into the mechanisms of tissue dependencies in the context of preterm labor and indicate that targeting the NLRP3 pathway could prevent future adverse perinatal outcomes. Understanding how the NLRP3 inflammasome participates in molecular and biochemical pathways in terms of parturition and how to control excessive NLRP3 inflammasome activation is essential for the identification of new targets for the treatment of reproductive dysfunction. It seems that administering MCC950 to patients could inhibit the development of the inflammatory reaction occurring during cervical ripening in preterm labor. Taking into account the huge contribution of the inflammatory reaction to preterm birth, it seems that inhibiting one of its components, i.e., activation of the NLRP3 inflammasome, may contribute to inhibiting preterm birth, thus reducing neonatal complications. However, this interesting topic still requires more research to develop the mechanism-specific and safe treatments for pregnancy disorders.

## 7. Conclusions

Taking all of the above into consideration, we believe that the NLRP3 inflammasome holds a very important place in cervical biology. By participating in most biochemical pathways, it seems that the NLRP3 inflammasome may be responsible for mediating, regulating, and ensuring the proper functioning of all biochemical and molecular pathways that take place in the cervical tissue during its maturation. A comprehensive understanding of cervical biology seems to be crucial in the context of developing new therapeutic options to manage severe adverse perinatal complications such as preterm parturition. Despite our deep knowledge of the biochemical and molecular events occurring during the cervical ripening process, the hunt for the factors that trigger and modulate this process persists. An extensive understanding of the rules of cervical ripening seems to be pivotal in terms of labor induction. Greater understanding could provide us with resources to help women avoid dysfunctional parturition.
